# Uniform asymptotics for ruin probabilities in a two-dimensional nonstandard renewal risk model with stochastic returns

**DOI:** 10.1186/s13660-018-1913-6

**Published:** 2018-11-19

**Authors:** Yinghua Dong, Dingcheng Wang

**Affiliations:** 1 College of Mathematics and Statistics, Nanjing University of Information Science and Technology, Nanjing, China; 2 School of Mathematical Science, University of Electric Science and Technology of China, Chengdu, China

**Keywords:** 62P05, 60G50, 26A12, Uniform asymptotic formulas, Ruin probabilities, Two-dimensional risk model, Stochastic returns, Dominated-variation distributions

## Abstract

In this paper, we consider a two-dimensional nonstandard renewal risk model with stochastic returns, in which the two lines of claim sizes form a sequence of independent and identically distributed random vectors following a bivariate Sarmanov distribution, and the two claim-number processes satisfy a certain dependence structure. When the two marginal distributions of the claim-size vector belong to the intersection of the dominated-variation class and the class of long-tailed distributions, we obtain uniform asymptotic formulas of finite-time and infinite-time ruin probabilities.

## Introduction

In this paper, we study a two-dimensional nonstandard renewal risk model with stochastic returns, in which an insurer simultaneously operates two kinds of insurance businesses. The claim sizes $\{(X,Y), (X_{i},Y_{i}), i\geq1\}$ form a sequence of independent and identically distributed (i.i.d.) and nonnegative random vectors, whose marginal distribution functions are denoted by $F(x)$ and $G(y)$ on $[0,\infty)$, respectively. Suppose that $(X,Y)$ follows a bivariate Sarmanov distribution of the following form:

1.1 $$ P(X\in du, Y\in dv)= \bigl(1+\theta\varphi_{1}(u)\varphi _{2}(v) \bigr)F(du)G(dv),\quad u\geq0, v\geq0, $$

where the kernels $\varphi_{1}(u)$ and $\varphi_{2}(v)$ are two functions and the parameter *θ* is a real constant satisfying

$$ E\varphi_{1}(X)=E\varphi_{2}(Y)=0, $$

and

$$ 1+\theta\varphi_{1}(u)\varphi_{2}(v)\geq0, \quad\text{for all } u \in D_{X}, v\in D_{Y}, $$

where $D_{X}=\{u \geq0: P(X\in(u-\delta, u+\delta))>0\text{ for all }\delta>0\}$ and $D_{Y}=\{v\geq0: P(Y\in(v-\delta, v+\delta))>0\text{ for all }\delta>0\}$. Clearly, if $\theta=0$ or $\varphi _{1}(u)\equiv0$, $u\in D_{X}$, or $\varphi_{2}(v)\equiv0$, $v\in D_{Y}$, then *X* and *Y* are independent. So we say that a random vector $(X,Y)$ follows a proper bivariate Sarmanov distribution, if the parameter $\theta\neq0$, and the kernels $\varphi_{1}(u)$ and $\varphi_{2}(v)$ are not identical to 0 in $D_{X}$ and $D_{Y}$, respectively. For more details of multivariate Sarmanov distributions, the read is referred to Lee [19] and Kotz et al. [18].

The Sarmanov family includes Falie–Gumbel–Morgenstern (FGM) distributions as special cases. For the FGM family, Schucany et al. [26] showed that both of the ranges of correlation coefficients and rank correlation coefficients are limited to $(-1/3, 1/3)$, and the Kendall *τ* coefficient equals $2/3$ of the rank correlation coefficient. The correlation coefficients of the Sarmanov family can attain a much wider range than those of the FGM family. Moreover, the range of correlation coefficients depends on marginal distributions. For example, for uniform and normal marginals, Shubina and Lee [27] proved that the ranges of correlation coefficients are $[-3/4, 3/4]$ and $[-2/\pi, 2/\pi]$, respectively. Shubina and Lee [27] and Huang and Lin [15] constructed some Sarmanov distributions, for which the correlation coefficients approach 1. For the Sarmanov family, Shubina and Lee [27] demonstrated that the range of rank correlation coefficients is $(-3/4, 3/4)$, while the range of Kendall *τ* coefficients is $(-1/2, 1/2)$. For simplicity, we assume that $\lim_{u\rightarrow\infty}\varphi_{1}(u)=d_{1}$ and $\lim_{v\rightarrow \infty}\varphi_{1}(v)=d_{2}$.

Let $c_{i}(t)$ represent the probability density function of premium income for the *i*th kind of insurance business at time *t*. Suppose that there is a positive constant *M* such that $0\leq c_{i}(t)\leq M$, $i=1,2$.

In risk theory, some publications suppose that two kinds of businesses share a common claim-number process or the two claim-number processes are mutually independent. It should be noted that these assumptions are made mainly for mathematical tractability. In reality, the claim-number processes of different insurance businesses are not always the same but closely dependent. We refer the reader to Ambagaspitiya [1] for details. Hence, establishing a bivariate risk model with a certain dependence structure between the two claim-number processes become more and more imperative. In this paper, let $\{\tau_{k}, k\geq1\}$ and $\{ \eta_{k}, k\geq1\}$ denote the arrival times of two kinds of successive claims, respectively. Suppose $\tau_{0}=0$ and $\eta _{0}=0$. We assume that $\{(\tau_{k}-\tau_{k-1}, \eta_{k}-\eta _{k-1}), k\geq1\}$ form another sequence of i.i.d. random vectors such that $\{(M(t), N(t)), t\geq0\}$ is a bivariate renewal process. Denote

$$ \lambda(u,v)=\sum_{i=1}^{\infty}\sum _{j=1}^{\infty}P(\tau_{i}\leq u, \eta_{j}\leq v). $$

Then $\lambda(u,v)$ is called a renewal function of the above bivariate renewal process.

In addition, when $\{(M(t), N(t)), t\geq0\}$ is a bivariate renewal process, it is easy to see that both $\{M(t), t\geq0\}$ and $\{N(t), t\geq0\}$ are one-dimensional renewal processes, and their renewal functions are denoted by $\lambda_{1}(t)$ and $\lambda_{2}(t)$, respectively.

Denote by *Λ* the set of all *t* for which $0<\lambda(t,t)\leq \infty$. Let $\underline{t}=\inf\{t:P(\tau_{1}\leq t, \eta _{1}\leq t)>0\}$. Then it is clear that

$$ \varLambda= \textstyle\begin{cases} [\underline{t}, \infty]& \text{if } P(\tau_{1}\leq \underline{t}, \eta_{1}\leq\underline{t})>0, \\ (\underline{t}, \infty] &\text{if } P(\tau_{1}\leq\underline{t}, \eta _{1}\leq\underline{t} )=0. \end{cases} $$

For more details of a bivariate renewal process, we refer the reader to Hunter [16]. Let $\varLambda_{T}=\varLambda\cap(0,T]$.

In addition, it is easy to get

$$ \lambda_{1}(t)=\sum_{i=1}^{\infty}P( \tau_{i}\leq t) \quad\text{and} \quad\lambda_{2}(t)=\sum _{j=1}^{\infty}P(\eta_{j}\leq t). $$

Suppose that the price processes of the investment portfolios for two kinds of insurance businesses are modeled by two geometric Lévy processes $\{e^{R_{1}(t)}, t\geq0\}$ and $\{e^{R_{2}(t)}, t\geq0\}$, where $\{R_{1}(t), t\geq0\}$ and $\{R_{2}(t), t\geq0\}$ are two Lévy processes which starts from 0, have independent and stationary increments, and are stochastically continuous. For any $i=1,2$, let $\{ R_{i}(t), t\geq0\}$ be a real-valued Lévy process with Lévy triplet $(r_{i}, \sigma_{i}, \rho_{i})$, where $-\infty< r_{i}<\infty $ and $\sigma_{i}>0$ are constants, and $\rho_{i}$ is a measure supported on $(-\infty, \infty)$, satisfying $\rho_{i}(0)=0$ and $\int_{(-\infty, \infty)}(y^{2}\wedge1)\rho_{i}(dy)<\infty$. According to Proposition 3.14 of Cont and Tankov [5], if $\int _{|y|\geq1}e^{zy}\rho_{i}(dy)<\infty$ for $z\in(-\infty, \infty)$, then the Laplace exponent for $\{R_{i}(t), t\geq0\}$ is defined as

$$ \varPhi_{i}(z)=\log Ee^{zR_{i}(1)},\quad z\in(-\infty, \infty), $$

where

$$ \varPhi_{i}(z)=\frac{1}{2}\sigma_{i}^{2}z^{2}+r_{i}z+ \int_{(-\infty, \infty)} \bigl(e^{zy}-1-zy\mathbf{1}_{(-1,1)}(y) \bigr)\rho_{i}(dy)< \infty. $$

Let

$$ \phi_{i}(z)=\varPhi_{i}(-z)=\frac{1}{2} \sigma_{i}^{2}z^{2}-r_{i}z+ \int _{(-\infty, \infty)} \bigl(e^{-zy}-1+zy\mathbf{1}_{(-1,1)}(y) \bigr)\rho _{i}(dy)< \infty. $$

Then, for all $t\geq0$ and *z* satisfying $\int_{|y|\geq1}e^{zy}\rho _{i}(dy)<\infty$, $Ee^{zR_{i}(t)}=e^{t\phi_{i}(-z)}<\infty$. Further, since $\phi_{i}(0)=0$, by the two expressions above, we can prove that $\phi_{i}(z)$ is convex in *z* for which $\phi_{i}(z)$ is finite. Since $\phi _{i}(0)=0$, for some $\beta^{*}>0$, $\phi_{i}(\beta^{*})<0$ means that $\phi_{i}(z)<0$ for all $z\in(0, \beta^{*}]$. For the general theory of Lévy processes, we refer the reader to Cont and Tankov [5] and Sato [25].

For two-dimensional risk models, some authors suppose that the insurance company invests the surpluses of two kinds of insurance businesses in one portfolio; see Fu and Ng [10], Li [20] and Guo et al. [14]. But such an assumption is restrictive in applications. In fact, an insurer often invests the surpluses of different businesses into different portfolios in order to avoid risks.

Throughout this paper, we suppose that $\{(X_{i},Y_{i}), i\geq1\}$, $\{ (c_{1}(t), c_{2}(t)), t\geq0\}$, $\{R_{1}(t), t\geq0\}$, $\{R_{2}(t), t\geq0\}$ and $\{(M(t), N(t)), t\geq0\}$ are mutually independent.

Denote the initial capital vector by $(x,y)$. For any time $t\geq0$, the surplus process of the insurer can be described as

1.2 $$\left(\begin{array}{c} U_{1}(t)\\ U_{2}(t) \end{array}\right)=\left(\begin{array}{c} xe^{R_{1}(t)}\\ ye^{R_{2}(t)} \end{array}\right)+\left(\begin{array}{c} \int_{0}^{t}e^{R_{1}(t)-R_{1}(s)}c_{1}(s)\;ds\\ \int_{0}^{t}e^{R_{2}(t)-R_{2}(s)}c_{2}(s)\;ds \end{array}\right)-\left(\begin{array}{c} \sum_{i=1}^{M(t)}X_{i}e^{R_{1}(t)-R_{1}(\tau_{i})}\\ \sum_{j=1}^{N(t)}Y_{j}e^{R_{2}(t)-R_{2}(\eta_{j})} \end{array}\right).$$

Next we define two types of ruin times for the risk model (1.2) as follows:

$$\begin{aligned} T_{\max}=\inf \bigl\{ t\geq0: \max \bigl\{ U_{1}(t), U_{2}(t) \bigr\} < 0 \bigr\} \end{aligned}$$

and

$$\begin{aligned} T_{\min}=\inf \bigl\{ t\geq0:\min \bigl\{ U_{1}(t), U_{2}(t) \bigr\} < 0 \bigr\} . \end{aligned}$$

Then the corresponding ruin probabilities of the risk model (1.2) are defined by

$$\begin{aligned} \psi_{\max}(x,y;t)=P \bigl(T_{\max}\leq t| \bigl(U_{1}(0), U_{2}(0) \bigr)=(x,y) \bigr),\quad t\geq0, \end{aligned}$$

and

$$\begin{aligned} \psi_{\min}(x,y;t)=P \bigl(T_{\min}\leq t| \bigl(U_{1}(0), U_{2}(0) \bigr)=(x,y) \bigr),\quad t\geq0, \end{aligned}$$

respectively. $\psi_{\max}(x,y;t)$ denotes the probability that ruin occurs in both business lines over the time $(0,t]$, while $\psi_{\min}(x,y;t)$ represents the probability that ruin occurs in at least one business line over the time $(0,t]$.

In the recent years, the one-dimensional renewal risk model with stochastic returns has been widely investigated. We refer the reader to Klüppelberg and Kostadinova [17], Tang et al. [29], Dong and Wang [6], Dong and Wang [7], Guo and Wang [12], Guo and Wang [13], and Peng and Wang [24], among many others. So far few articles have been involved in a bivariate risk model with stochastic returns. For example, Fu and Ng [10] considered a two-dimensional renewal risk model with stochastic returns, in which the claim sizes for the same kind of insurance business are pairwise quasi-independent but the claim sizes of different kinds of insurance businesses are independent, and presented a uniform asymptotic formula only for the discounted aggregate claims. Li [20] considered a multi-dimensional renewal risk model, where there exists a certain dependence structure among claim sizes and their corresponding inter-arrival times. When the claim-size vector has a multi-dimensional regular variation distribution, the authors gave a uniform asymptotic formula for ruin probabilities over all the whole times. Guo et al. [14] studied another two-dimensional risk model with stochastic investment returns, where two lines of insurance businesses share a common claim-number process and their surpluses are invested into the same kind of risky asset, and the claim sizes of two kinds of insurance businesses and their common inter-arrival times correspondingly follow a three-dimensional Sarmanov distribution. When the marginal distributions of the claim-size vector belong to the regular variation class, the above reference presented uniform asymptotic formulas for the finite-time ruin probability. Fu and Ng [11] discussed a two-dimensional renewal risk model, in which there is a FGM structure between the claim sizes from two different lines of businesses, and showed uniform asymptotic formulas of the finite-time ruin probability, when the distributions of claim sizes belong to the intersection of the dominated varying class and the class of long-tailed distributions.

In the present paper, we investigate a bivariate renewal risk model with stochastic returns, where the claim sizes form a sequence of i.i.d. random vectors following a bivariate Sarmanov distribution and the price processes of investment portfolios are modeled by two geometric Lévy processes. When the two marginal distributions of the claim-size vector belong to the intersection of the dominated-variation class and the class of long-tailed distributions, we obtain uniform asymptotic formulas of the joint tail probability of the discounted aggregate claims and ruin probabilities for the risk model (1.2).

The rest of this paper is organized as follows. In Sect. 2, we recall some important distribution classes and give main results of this paper. In Sect. 3, we prepare some necessary lemmas. In Sect. 4, we prove the two theorems.

## Preliminaries and main results

This paper is concerned with heavy-tailed distributions, so we first introduce some related subclasses of heavy-tailed distributions, which can be found in Embrechts et al. [8], Bingham et al. [2], and Cline and Samorodnitsky [4]. Let *H* be a distribution and write $\overline{H}(x)=1-H(x)$. We assume that $\overline{H}(x)>0$ holds for all $x>0$. We say that a distribution *H* on $[0, \infty)$ belongs to the class of long-tailed distributions, denoted by $\mathcal{L}$, if for any $u>0$,

$$\begin{aligned} \lim_{x\rightarrow\infty} \frac{\overline{H}(x+u)}{\overline{H}(x)}=1. \end{aligned}$$

A distribution *H* on $[0, \infty)$ is said to belong to the dominated-varying-tailed class $\mathcal{D}$, if for all $0< u<1$,

$$\begin{aligned} \limsup_{x\rightarrow\infty} \frac{\overline{H}(ux)}{\overline{H}(x)}< \infty. \end{aligned}$$

We say that a distribution *H* on $[0,\infty)$ belongs to the regular variation class, if there is some *α*, $0<\alpha<\infty$, such that, for all $u>0$,

$$\begin{aligned} \lim_{x\rightarrow\infty} \frac{\overline{H}(ux)}{\overline{H}(x)}=u^{-\alpha}. \end{aligned}$$

In this case, we denote $H\in\mathcal{R_{-\alpha}}$ and use $\mathcal{R}$ to denote the union of all $\mathcal{R_{-\alpha}}$ over the range $0<\alpha<\infty$. It is well known that $\mathcal{R}\subset\mathcal{D}\cap\mathcal {L}$ and the inclusion is proper.

We introduce two indices of any distribution *H*. Denote

$$\begin{aligned} J_{H}^{+}=-\lim_{y\rightarrow\infty}\frac{\log\overline {H}_{*}(y)}{\log y}\quad \text{and}\quad J_{H}^{-}=-\lim_{y\rightarrow\infty} \frac{\log\overline{H}^{*}(y)}{\log y}. \end{aligned}$$

Following Tang and Tsitsiashvili [28], we call $J_{H}^{+}$ and $J_{H}^{-}$ the upper and lower Matuszewska indices of *H*.

Hereafter, all limit relationships are for $\min(x,y)\rightarrow \infty$ unless stated otherwise. For two positive functions $a(x,y)$ and $b(x,y)$, we write $a(x,y)\lesssim b(x,y)$ if $\limsup_{\min (x,y)\rightarrow\infty} a(x,y)/ b(x,y)\leq1$, write $a(x,y)\gtrsim b(x,y)$ if $\liminf a(x,y)/b(x,y)\geq1$, write $a(x,y)\thicksim b(x,y)$ if $a(x,y)\lesssim b(x,y)$ and $a(x,y)\gtrsim b(x,y)$, and write $a(x,y)=o(b(x,y))$ if $\lim_{\min(x,y)\rightarrow\infty} a(x,y)/b(x,y)=0$. Furthermore, for two positive ternary functions $a(\cdot,\cdot;t)$ and $b(\cdot,\cdot;t)$, we say that the asymptotic relation $a(x,y;t)\sim b(x,y;t)$ holds uniformly for *t* in a nonempty set Δ if

$$ \lim_{\min(x,y)\rightarrow\infty}\sup_{t\in \Delta} \biggl\vert \frac{a(x,y;t)}{b(x,y;t)}-1 \biggr\vert =0. $$

Clearly, the asymptotic relation $a(x,y;t)\sim b(x,y;t)$ holds uniformly for $t\in\Delta$ if and only if

$$ \limsup_{\min(x,y)\rightarrow\infty}\sup_{t\in \Delta}\frac{a(x,y;t)}{b(x,y;t)} \leq1 \quad\text{and}\quad \liminf_{\min(x,y)\rightarrow\infty}\inf_{t\in \Delta} \frac{a(x,y;t)}{b(x,y;t)}\geq1, $$

which means that both $a(x,y;t)\lesssim b(x,y;t)$ and $a(x,y;t)\gtrsim b(x,y;t)$ hold uniformly for $t\in \Delta$.

Now we are in a position to state our main results. We first present a uniform asymptotic formula of the joint tail probability of two discounted aggregate claims. Then we establish uniform asymptotic formulas of ruin probabilities.

### Theorem 2.1

*Consider the risk model* (1.2). *Let* $\{(X,Y),(X_{k},Y_{k}), k\geq1\}$ *be i*.*i*.*d*. *random vectors following a bivariate Sarmanov distribution of the form* (1.1), *where* $\lim_{x\rightarrow\infty}\phi_{i}(x)=d_{i}$ *for* $i=1,2$. *Suppose that the distributions of* *X* *and* *Y* *satisfy* $F\in\mathcal {D}\cap\mathcal{L}$ *and* $G\in\mathcal{D}\cap\mathcal{L}$ *with* $J_{F}^{-}>0$ *and* $J_{G}^{-}>0$. *If* $\phi_{i}(\beta_{i})<0, i=1,2$, *for some* $\beta_{1}>J_{F}^{+}$ *and* $\beta_{2}>J_{G}^{+}$, *then uniformly for all* $t\in\varLambda$

2.1 $$\begin{aligned} &P \Biggl(\sum_{i=1}^{M(t)}X_{i}e^{-R_{1}(\tau_{i})}>x, \sum_{j=1}^{N(t)}Y_{j}e^{-R_{2}(\eta_{j})}>y \Biggr) \\ &\quad\thicksim \int_{0}^{t} \int_{0}^{t}P \bigl(X^{*}e^{-R_{1}(u)}>x \bigr)P \bigl(Y^{*}e^{-R_{2}(v)}>y \bigr) \lambda(du, dv) \\ &\qquad{}+ \theta d_{1}\,d_{2}\sum_{i=1}^{\infty } \int_{0}^{t} \int_{0}^{t}P \bigl(X^{*}e^{-R_{1}(u)}>x \bigr)P \bigl(Y^{*}e^{-R_{2}(v)}>y \bigr) P(\tau_{i}\in du, \eta_{i}\in dv), \end{aligned}$$

*where* $X^{*}$ *and* $Y^{*}$ *are two independent nonnegative random variables with distributions* *F* *and* *G*, *respectively*.

### Theorem 2.2

*Under the conditions of Theorem *2.1,

2.2 $$\begin{aligned} \psi_{\max}(x,y;t) \thicksim{}& \int_{0}^{t} \int _{0}^{t}P \bigl(X^{*}e^{-R_{1}(u)}>x \bigr)P \bigl(Y^{*}e^{-R_{2}(v)}>y \bigr) \lambda(du, dv)+\theta d_{1}d_{2} \\ & {}\times\sum_{i=1}^{\infty} \int_{0}^{t} \int _{0}^{t}P \bigl(X^{*}e^{-R_{1}(u)}>x \bigr)P \bigl(Y^{*}e^{-R_{2}(v)}>y \bigr) P(\tau_{i}\in du, \eta_{i}\in dv) \end{aligned}$$

*holds uniformly for all* $t\in\varLambda$. *In addition*, *for any* $T\in\varLambda$,

2.3 $$\begin{aligned} \psi_{\min}(x,y;t) \thicksim \int _{0}^{t}P \bigl(Xe^{-R_{1}(u)}>x \bigr) \lambda_{1}(u)+ \int_{0}^{t}P \bigl(Ye^{-R_{2}(v)}>y \bigr) \,d \lambda_{2}(v) \end{aligned}$$

*holds uniformly for all* $t\in\varLambda_{T}$. *In particular*,

$$\begin{aligned} &\psi_{\max}(x,y;\infty) \\ &\quad \thicksim \int_{0}^{\infty } \int_{0}^{\infty}P \bigl(X^{*}e^{-R_{1}(u)}>x \bigr)P \bigl(Y^{*}e^{-R_{2}(v)}>y \bigr) \lambda(du, dv) \\ &\qquad{}+ \theta d_{1}d_{2}\sum_{i=1}^{\infty } \int_{0}^{\infty} \int_{0}^{\infty }P \bigl(X^{*}e^{-R_{1}(u)}>x \bigr)P \bigl(Y^{*}e^{-R_{2}(v)}>y \bigr) P(\tau_{i}\in du, \eta_{i}\in dv). \end{aligned}$$

*By the definition of the regular variation class and Theorem *2.2, *we easily obtain the following corollary*.

### Corollary 2.1

*Consider the risk model* (1.2). *Suppose that the conditions of Theorem *2.1 *are satisfied*. *Further*, *if the distributions of* *X* *and* *Y* *satisfy* $F\in\mathcal{R_{-\alpha}}$ *and* $G\in\mathcal{R_{-\alpha}}$ *with* $0<\alpha<\infty$, *then*

$$\begin{aligned} \psi_{\max}(x,y;t) \thicksim{}& \int_{0}^{t} \int_{0}^{t}e^{u \phi _{1}(\alpha)+v \phi_{2}(\alpha)} \lambda(du, dv) \overline{F}(x)\overline{G}(y) \\ &{}+ \theta d_{1}d_{2}\sum_{i=1}^{\infty} \int_{0}^{t} \int_{0}^{t}e^{u \phi _{1}(\alpha)+v \phi_{2}(\alpha)} P(\tau_{i} \in du, \eta_{i}\in dv)\overline{F}(x)\overline{G}(y) \end{aligned}$$

*holds uniformly for all* $t\in\varLambda$. *In addition*, *for any* $T\in \varLambda$,

$$\begin{aligned} \psi_{\min}(x,y;t) \thicksim \int_{0}^{t}e^{u \phi_{1}(\alpha )}\,d \lambda_{1}(u)\overline{F}(x)+ \int_{0}^{t}e^{v \phi_{2}(\alpha)} \,d \lambda_{2}(v)\overline{G}(y) \end{aligned}$$

*holds uniformly for all* $t\in\varLambda_{T}$.

## Some lemmas

The first lemma is from Lemma 2.19 of Foss et al. [9].

### Lemma 3.1

*If* $H\in\mathcal{L}$, *then there exists a slowly varying function* $h(x)$ *satisfying* $0< h(x)\rightarrow\infty $, $h(x)/x\rightarrow0$, *such that*

$$\begin{aligned} \lim_{x\rightarrow\infty}\frac{\overline{H}(x\pm h(x))}{\overline {H}(x)}=1. \end{aligned}$$

The lemma below is due to Proposition 1.1 of Yang and Wang [31].

### Lemma 3.2

*Suppose that* $(X,Y)$ *follows a proper bivariate Sarmanov distribution of the form* (1.1). *Then there exist two positive constants* $b_{1}$ *and* $b_{2}$ *such that* $|\varphi _{1}(u)|\leq b_{1}$ *for all* $u\in D_{X}$ *and* $|\varphi_{2}(v)|\leq b_{2}$ *for all* $v\in D_{Y}$.

The following lemma is a combination of Proposition 2.2.1 of Bingham et al. [2] and Lemma 3.5 of Tang and Tsitsiashvili [28].

### Lemma 3.3

*For a distribution* *H* *on* $[0,\infty)$, *the following assertions hold*:

1. *if* $H\in\mathcal{D}$, *then*, *for any* $\alpha< J_{H}^{-}$ *and* $\beta>J_{H}^{+}$, *there are positive numbers* $C_{i}$ *and* $D_{i}, i=1,2$, *such that*
  $$\begin{aligned} \frac{\overline{H}(y)}{\overline{H}(x)}\geq C_{1} \biggl(\frac{x}{y} \biggr)^{\alpha} \quad \textit{for all } x\geq y\geq D_{1} \end{aligned}$$
  *and*
  $$\begin{aligned} \frac{\overline{H}(y)}{\overline{H}(x)}\leq C_{2} \biggl(\frac{x}{y} \biggr)^{\beta} \quad\textit{for all } x\geq y\geq D_{2}; \end{aligned}$$
2. *if* $H\in\mathcal{D}$, *then*
  $$\begin{aligned} x^{-\beta}=o \bigl(\overline{H}(x) \bigr) \quad\textit{for all } \beta>J_{H}^{+}. \end{aligned}$$

The following lemma is a restatement of Lemma 4.1.2 of Wang and Tang [30].

### Lemma 3.4

*Let* *X* *and* *ξ* *be two independent random variables*, *where* *X* *is distributed by* $F\in\mathcal{D}\cap \mathcal{L}$ *and* *ξ* *is nonnegative and non*-*degenerate at* 0 *satisfying* $E\xi ^{p}<\infty$ *for some* $p>J_{F}^{+}$. *Then the distribution of the product* *ξX* *belongs to the class* $\mathcal{D}\cap\mathcal{L}$ *and* $P(\xi X>x)\asymp P(X>x)$.

### Remark 1

*Suppose that* *X* *and* *Y* *are two nonnegative random variables with distributions* $F\in D\cap L$ *and* $G\in D\cap L$, *and* $\phi_{i}(\beta_{i})<0$, $i=1,2$, *for some* $\beta _{1}>J_{F}^{+}$ *and* $\beta_{2}>J_{G}^{+}$. *Then*, *by Lemma *3.4, *we can prove both* $Xe^{-R_{1}(s)}$ *and* $Ye^{-R_{2}(w)}$ *belong to* $\mathcal {D}\cap\mathcal{L}$ *for any* $s>0$ *and* $w>0$. *Hence*, *by Lemma *3.1 *above and Proposition *2.20(i) *of Foss et al*. [9], *there exists a positive function* $h(x)$ *satisfying* $h(x)\rightarrow\infty$, $h(x)/x\rightarrow0$, *such that*

3.1 $$\begin{aligned} \lim_{x\rightarrow\infty}\frac{P (Xe^{-R_{1}(s)}>x-h(x) )}{P (Xe^{-R_{1}(s)}>x )}=1 \end{aligned}$$

*and*

3.2 $$\begin{aligned} \lim_{y\rightarrow\infty}\frac{P (Ye^{-R_{2}(w)}>y-h(y ) )}{P (Ye^{-R_{2}(w)}>y )}=1. \end{aligned}$$

The lemma below can be derived from Lemma 5 of Chen et al. [3].

### Lemma 3.5

*Let* $\{X_{i}, 1\leq i\leq n\}$ *be a sequence of independent random variables with common distribution* $F\in \mathcal{D}\cap\mathcal{L}$. *Suppose that* $\{\xi_{i}, 1\leq i\leq n\}$ *is another sequence of nonnegative and non*-*degenerate at* 0 *random variables satisfying* $E\xi_{i}^{p}<\infty$ *for some* $p>J_{F}^{+}$. *If* $\{\xi_{i}, 1\leq i\leq n\}$ *is independent of* $\{ X_{i}, 1\leq i\leq n\}$, *then*

$$\begin{aligned} \lim_{x\wedge y\rightarrow\infty}\frac{P(\xi_{i}X_{i}>x, \ \xi _{j}X_{j}>y)}{P(\xi_{j}X_{j}>y)}=0 \end{aligned}$$

*holds for all* $1\leq i\neq j\leq n$.

The following lemma gives an important property of bivariate Sarmanov distributions and it is also interesting by itself.

### Lemma 3.6

*Suppose that* $(X,Y)$ *follows a bivariate Sarmanov distribution* (1.2) *with* $\lim_{x\rightarrow\infty }\varphi_{i}(x)=d_{i}$ *for* $i=1,2$. *Then*

$$\begin{aligned} P(X>x, Y>y)\thicksim(1+\theta d_{1}d_{2})\overline{F}(x) \overline {G}(y). \end{aligned}$$

### Proof

By (1.1),

$$\begin{aligned} P(X>x, Y>y)= \int_{x}^{\infty} \int_{y}^{\infty} \bigl(1+\theta\varphi_{1}(u) \varphi _{2}(v) \bigr)F(du)G(dv)\thicksim(1+\theta d_{1}d_{2})\overline{F}(x) \overline {G}(y). \end{aligned}$$

 □

By Lemmas 3.3(2), 3.5 and 3.6, the following lemma can be derived from Lemma 3(ii) of Li [21].

### Lemma 3.7

*Let* $(X,Y)$ *follow a bivariate Sarmanov distribution of the form* (1.1) *with* $\lim_{x\rightarrow\infty }\varphi_{i}(x)=d_{i}$ *for* $i=1,2$. *Suppose that* $\phi_{i}(\beta _{i})<0, i=1,2$, *for some* $\beta_{1}>J_{F}^{+}$ *and* $\beta_{2}>J_{G}^{+}$. *If the distributions of* *X* *and* *Y* *satisfy* $F\in\mathcal{D}\cap \mathcal{L}$ *and* $G\in\mathcal{D}\cap\mathcal{L}$, *then*, *for any* $s>0$ *and* $w>0$,

$$\begin{aligned} &P \bigl(Xe^{-R_{1}(s)}>x-h(x), Ye^{-R_{2}(w)}>y-h(y) \bigr) \\ &\quad \thicksim (1+\theta d_{1}d_{2})P \bigl(X^{*}e^{-R_{1}(s)}>x \bigr)P \bigl(Y^{*}e^{-R_{2}(w)}>y \bigr), \end{aligned}$$

*where* $h(x)$ *is defined as in* (3.1) *and* (3.2).

In view of Theorem 2.1 in Li [21] and Lemma 3.7, we arrive at the following lemma.

### Lemma 3.8

*Let* $\{(X,Y), (X_{i}, Y_{i}), i\geq1\} $ *be a sequence of i*.*i*.*d*. *nonnegative random vectors following a bivariate Sarmanov distribution of the form* (1.1). *Suppose that* $\phi_{i}(\beta_{i})<0, i=1,2$, *for some* $\beta_{1}>J_{F}^{+}$ *and* $\beta_{2}>J_{G}^{+}$. *If the distributions of* *X* *and* *Y* *satisfy* $F\in\mathcal{D}\cap\mathcal{L}$ *and* $G\in\mathcal{D}\cap \mathcal{L}$, *then*, *for any fixed* $m\geq1$ *and* $n\geq1$, *uniformly for all* $0< s_{i}\leq t$, $0< t_{i}\leq t$ *and* $t\in\varLambda_{T}$,

$$\begin{aligned} &P \Biggl(\sum_{i=1}^{m}X_{i}e^{-R_{1}(s_{i})}>x, \sum_{j=1}^{n}Y_{j}e^{-R_{2}(t_{j})}>y \Biggr) \\ &\quad \thicksim \sum_{i=1}^{m}\sum _{j=1}^{n}P \bigl(X_{i}e^{-R_{1}(s_{i})}>x, Y_{j}e^{-R_{2}(t_{j})}>y \bigr). \end{aligned}$$

Following the proof of Theorem 1.1 in Liu and Zhang [22] with some modifications, we can get the lemma below.

### Lemma 3.9

*Let* $\{(X, Y), (X_{i},Y_{i}), i\geq1\} $ *be a sequence of i*.*i*.*d*. *nonnegative random vectors following a bivariate Sarmanov distribution of the form* (1.2). *Suppose that the distributions of* *X* *and* *Y* *satisfy* $F\in\mathcal{D}\cap\mathcal {L}$ *and* $G\in\mathcal{D}\cap\mathcal{L}$ *with* $0< J_{F}^{-}\leq J_{F}^{+}<\infty$ *and* $0< J_{G}^{-}\leq J_{G}^{+}<\infty$. *Assume that* $\{\xi_{i}, i\geq1\}$ *and* $\{\zeta_{j}, j\geq1\}$ *are another two sequences of nonnegative random variables*, *and that there exist* $p_{1}$, $p_{2}$ *and* *p* *satisfying* $0< p_{1}< J_{F}^{-}$, $0< p_{2}< J_{G}^{-}$ *and* $p>\max\{J_{F}^{+}, J_{G}^{+}\}$ *such that*

$$\begin{aligned} &\sum_{i=1}^{\infty} \bigl(E\xi_{i}^{p_{1}} \bigr)^{\mathbf {1}_{(J_{F}^{+}< 1)}+\frac{1}{p}\mathbf{1}_{(J_{F}^{+}\geq1)}}< \infty , \qquad\sum_{i=1}^{\infty} \bigl(E\xi_{i}^{p} \bigr)^{\mathbf {1}_{(J_{F}^{+}< 1)}+\frac{1}{p}\mathbf{1}_{(J_{F}^{+}\geq1)}}< \infty, \\ &\sum_{j=1}^{\infty} \bigl(E\zeta_{j}^{p_{2}} \bigr)^{\mathbf {1}_{(J_{G}^{+}< 1)}+\frac{1}{p}\mathbf{1}_{(J_{G}^{+}\geq1)}}< \infty ,\qquad \sum_{j=1}^{\infty} \bigl(E\zeta_{j}^{p} \bigr)^{\mathbf {1}_{(J_{G}^{+}< 1)}+\frac{1}{p}\mathbf{1}_{(J_{G}^{+}\geq1)}}< \infty. \end{aligned}$$

*Then*

$$\begin{aligned} P \Biggl(\sum_{i=1}^{\infty} \xi_{i}X_{i}>x, \sum_{j=1}^{\infty } \zeta_{j}Y_{j}>y \Biggr)\thicksim \sum _{i=1}^{\infty}\sum_{j=1}^{\infty}P( \xi_{i}X_{i}>x, \zeta _{j}Y_{j}>y). \end{aligned}$$

### Remark 2

*For the geometric Lévy process* $\{ e^{R_{1}(t)}, t\geq0\}$, *when* $J_{F}^{+}\leq1$, *there exists some* $\beta_{1}$ *satisfying* $\beta_{1}>J_{F}^{+}$ *and* $\phi(\beta _{1})<0$, *such that*, *for any* $0< p_{1}<\beta_{1}$,

$$\begin{aligned} \sum_{i=1}^{\infty}Ee^{-p_{1}R_{1}(\tau_{i})}= \sum _{i=1}^{\infty} \int_{0}^{\infty}e^{s\phi_{1}(p_{1})}P(\tau _{i} \leq s)=\frac{Ee^{\tau_{1}\phi_{1}(p_{1})}}{1-Ee^{\tau_{1}\phi _{1}(p_{1})}}< \infty. \end{aligned}$$

*When* $J_{F}^{+}>1$, *we can choose some* *p* *satisfying* $\beta _{1}>p>J_{F}^{+}$. *Likewise*,

$$\begin{aligned} \sum_{i=1}^{\infty} \bigl(Ee^{-pR_{1}(\tau_{i})} \bigr)^{1/p}= \sum_{i=1}^{\infty} \bigl(Ee^{\tau_{i}\phi_{1}(p)} \bigr)^{1/p}= \sum_{i=1}^{\infty} \bigl(Ee^{\tau_{1}\phi_{1}(p)} \bigr)^{i/p}< \infty. \end{aligned}$$

*Similar results hold for the geometric Lévy process* $\{ e^{R_{2}(t)}, t\geq0\}$. *Hence*, *by Lemma *3.9,

$$\begin{aligned} &P \Biggl(\sum_{i=1}^{\infty}X_{i}e^{-R_{1}(\tau_{i})}>x, \sum_{j=1}^{\infty}Y_{j}e^{-R_{2}(\eta_{j})}>y \Biggr) \\ &\quad \thicksim \sum_{i=1}^{\infty}\sum _{j=1}^{\infty}P \bigl(X_{i}e^{-R_{1}(\tau _{i})}>x, Y_{j}e^{-R_{2}(\eta_{j})}>y \bigr). \end{aligned}$$

For simplicity, for $t>0$, denote $\varOmega_{1}(t)=[0,t]\times(t,\infty )$, $\varOmega_{2}(t)=(t,\infty)\times[0,t]$ and $\varOmega _{3}(t)=(t,\infty)\times(t,\infty)$. By a simply calculation, we can obtain the following lemma.

### Lemma 3.10

*Under the conditions of Theorem *2.1, *for any* $k=1,2,3$, *the following assertions hold*:

3.3 $$\begin{aligned} \lim_{t\rightarrow\infty}\limsup_{\min(x,y)\rightarrow\infty } \frac{\int\int_{\varOmega_{k}(t)}P(X^{*}e^{-R_{1}(u)}>x)P( Y^{*}e^{-R_{2}(v)}>y)\lambda(du,dv)}{\int_{0}^{t}\int _{0}^{t}P(X^{*}e^{-R_{1}(u)}>x)P(Y^{*}e^{-R_{2}(v)}>y)\lambda (du,dv)}=0 \end{aligned}$$

*and*

3.4 $$\begin{aligned} &\lim_{t\rightarrow\infty}\limsup_{\min(x,y)\rightarrow\infty }\sup _{i\geq1}\frac{\int\int_{\varOmega _{k}(t)}P(X^{*}e^{-R_{1}(u)}>x)P( Y^{*}e^{-R_{2}(v)}>y)P(\tau_{i}\in du, \eta_{i}\in dv)}{\int_{0}^{t}\int _{0}^{t}P(X^{*}e^{-R_{1}(u)}>x)P(Y^{*}e^{-R_{2}(v)}>y)P(\tau_{i}\in du, \eta_{i}\in dv)} \\ &\quad =0. \end{aligned}$$

### Proof

It suffices to prove the first expression for $k=1$. By the proof of Lemma 4.3 in Tang et al. [29], we know that $E(e^{-p\inf_{0\leq u\leq t}R_{1}(u)})<\infty$ holds for $0< p<\beta _{1}$, and that $P(e^{-\sup_{0\leq u\leq t}R_{1}(u)}>\epsilon)>0$ holds for $0<\epsilon<1$. By Lemma 3.4,

$$\begin{aligned} &\lim_{t\rightarrow\infty}\limsup_{\min(x,y)\rightarrow\infty }\frac{\int\int_{\varOmega_{1}(t)}P(X^{*}e^{-R_{1}(u)}>x)P( Y^{*}e^{-R_{2}(v)}>y)\lambda(du,dv)}{\int_{0}^{t}\int _{0}^{t}P(X^{*}e^{-R_{1}(u)}>x)P(Y^{*}e^{-R_{2}(v)}>y)\lambda (du,dv)} \\ &\quad\leq \lim_{x\rightarrow\infty}\frac {P(X^{*}e^{-\inf_{0\leq u\leq t}R_{1}(u)}>x)}{P(X^{*}\epsilon >x)P(e^{-\sup_{0\leq u\leq t}R_{1}(u)}>\epsilon)} \\ &\qquad {}\times\lim_{t\rightarrow\infty}\lim_{y\rightarrow\infty}\frac {\int_{t}^{\infty}P(Y^{*}e^{-R_{2}(v)}>y)\,d\lambda_{2}(v)}{ \int_{0}^{t}P(Y^{*}e^{-R_{2}(v)}>y)\,d\lambda_{2}(v)}=0. \end{aligned}$$

In the same way, for $k=1,2,3$, (3.3) and (3.4) follow. □

In order to prove Theorem 2.2, we define ruin times for the two kinds of insurance businesses. Denote

$$\begin{aligned} \vartheta_{i}=\inf \bigl\{ t\geq0: U_{i}(t)< 0 \bigr\} ,\quad i=1,2. \end{aligned}$$

The following lemma plays an important role in proving Theorem 2.2.

### Lemma 3.11

*Under the conditions of Theorem *2.1, *we have*

3.5 $$\begin{aligned} P(\vartheta_{1}\leq t)\thicksim \int _{0}^{t}P \bigl(X^{*}e^{-R_{1}(u)}>x \bigr)\,d\lambda_{1}(u) \end{aligned}$$

*and*

3.6 $$\begin{aligned} P(\vartheta_{2}\leq t)\thicksim \int _{0}^{t}P \bigl(Y^{*}e^{-R_{2}(v)}>y \bigr)\,d\lambda_{2}(v) \end{aligned}$$

*hold uniformly for all* $t\in\varLambda$.

### Proof

In proving Theorem 1.2 of Fu and Ng [10], for $F\in\mathcal{D}\cap\mathcal{L}$, applying Theorem 1.1 of Liu and Zhang [22] instead of Theorem 2 of Yi et al. [32], we can arrive at

3.7 $$\begin{aligned} P \Biggl(\sum_{i=1}^{M(t)}X_{i}e^{-R_{1}(\tau_{i})}>x \Biggr)\thicksim \int_{0}^{t}P \bigl(X^{*}e^{-R_{1}(u)}>x \bigr)\,d\lambda_{1}(u) \end{aligned}$$

holds uniformly for all $t\in\varLambda$. Hence, it is clear that

3.8 $$\begin{aligned} P(\vartheta_{1}\leq t)\leq P \Biggl(\sum _{i=1}^{M(t)}X_{i}e^{-R_{1}(\tau_{i})}>x \Biggr) \thicksim \int _{0}^{t}P \bigl(X^{*}e^{-R_{1}(u)}>x \bigr)\,d\lambda_{1}(u) \end{aligned}$$

holds uniformly for all $t\in\varLambda$.

Next we turn to the proof of the asymptotic lower bound of (3.5). Since $F\in\mathcal{D}\cap\mathcal{L}$, according to Lemma 3.4, for any $0\leq u\leq t$, the distribution of $X^{*}e^{-R_{1}(u)}$ still belongs to $\mathcal{D}\cap\mathcal{L}$. By Remark 1, there exists some slowly varying function $l(x)$ satisfying $0< l(x)\rightarrow\infty$, $l(x)/x\rightarrow0$ such that, for any $0\leq u\leq t$,

3.9 $$\begin{aligned} \lim_{x\rightarrow\infty}\frac{P (X^{*}e^{-R_{1}(u)}>x+l(x) )}{P (X^{*}e^{-R_{1}(u)}>x )}=1. \end{aligned}$$

From Sect. 2.1 of Maulik and Zwart [23], we can see that $\int _{0}^{\infty}e^{-R_{1}(u)}\,du$ is light-tailed. Hence, by (3.7), (3.9) and Fatou’s lemma, uniformly for all $t\in\varLambda$,

3.10 $$\begin{aligned} P(\vartheta_{1}\leq t)&\geq P \Biggl(\sum _{i=1}^{M(t)}X_{i}e^{-R_{1}(\tau_{i})}-M \int_{0}^{\infty }e^{-R_{1}(u)}\,du>x \Biggr) \\ &\gtrsim P \Biggl(\sum_{i=1}^{M(t)} X_{i}e^{-R_{1}(\tau_{i})}>x+l(x) \Biggr) \\ &\gtrsim \int_{0}^{t}P \bigl(X^{*}e^{-R_{1}(u)}>x \bigr)\,d\lambda_{1}(u). \end{aligned}$$

A combination of (3.8) and (3.10) yields (3.5) holds uniformly for all $t\in\varLambda$. In the same way, we can prove that (3.6) also holds uniformly for all $t\in\varLambda$. □

## Proofs of main results

### Proof of Theorem 2.1

Choose some fixed positive integer *M*. Uniformly for all $t\in\varLambda_{T}$

4.1 $$\begin{aligned} &P \Biggl(\sum_{i=1}^{M(t)}X_{i}e^{-R_{1}(\tau_{i})}>x, \sum_{j=1}^{N(t)}Y_{j}e^{-R_{2}(\eta_{j})}>y \Biggr) \\ &\quad= \sum_{m=1}^{\infty}\sum _{n=1}^{\infty} P \Biggl(\sum _{i=1}^{m}X_{i}e^{-R_{1}(\tau_{i})}>x, \sum _{j=1}^{n}Y_{j}e^{-R_{2}(\eta_{j})}>y, M(t)=m, N(t)=n \Biggr) \\ &\quad= \Biggl(\sum_{m=1}^{M}\sum _{n=1}^{M}+\sum_{m=1}^{M} \sum_{n=M+1}^{\infty}+\sum _{m=M+1}^{\infty}\sum_{n=1}^{M}+ \sum_{m=M+1}^{\infty}\sum _{n=M+1}^{\infty} \Biggr) \\ & \qquad{}\times P \Biggl(\sum_{i=1}^{m}X_{i}e^{-R_{1}(\tau_{i})}>x, \sum_{j=1}^{n}Y_{j}e^{-R_{2}(\eta_{j})}>y, M(t)=m, N(t)=n \Biggr) \\ &\quad \equiv K_{1}(x,y;t)+K_{2}(x,y;t)+K_{3}(x,y;t)+K_{4}(x,y;t). \end{aligned}$$

We first consider $K_{1}(x,y;t)$. For $m\geq1$ and $n\geq1$, write $\varOmega^{(1)}(m)=\{0\leq s_{1}\leq\cdots\leq s_{m}\leq t, s_{m+1}>t\}$ and $\varOmega^{(2)}(n)=\{0\leq t_{1}\leq\cdots\leq t_{n}\leq t, t_{n+1}>t\}$. By Lemma 3.8, uniformly for all $t\in \varLambda_{T}$

$$\begin{aligned} &K_{1}(x,y;t) \\ &\quad \sim\sum_{m=1}^{M}\sum _{n=1}^{M}\sum_{i=1}^{m} \sum_{j=1}^{n} \int_{\varOmega^{(1)}(m)\times\varOmega ^{(2)}(n)}P \bigl(X^{*}e^{-R_{1}(s_{i})}>x, Y^{*}e^{-R_{2}(t_{j})}>y \bigr) \\ &\qquad{}\times P(\tau_{1}\in s_{1},\ldots, \tau_{m+1}\in s_{m+1}, \eta_{1}\in t_{1},\ldots, \eta_{n+1}\in t_{n+1}) \\ &\quad = \sum_{m=1}^{M}\sum _{n=1}^{M}\sum_{i=1}^{m} \sum_{j=1}^{n}P \bigl(X_{i}e^{-R_{1}(\tau_{i})}>x, Y_{j}e^{-R_{2}(\eta_{j})}>y, M(t)=m, N(t)=n \bigr). \end{aligned}$$

According to the above expression, uniformly for all $t\in\varLambda_{T}$,

$$\begin{aligned} &K_{1}(x,y;t) \\ &\quad \sim\sum_{m=1}^{\infty}\sum _{n=1}^{\infty}\sum_{i=1}^{m} \sum_{j=1}^{n}P \bigl(X_{i}e^{-R_{1}(\tau_{i})}>x, Y_{j}e^{-R_{2}(\eta_{j})}>y, M(t)=m, N(t)=n \bigr) \\ &\qquad{}-\sum_{m=1}^{\infty}\sum _{n=M+1}^{\infty }\sum_{i=1}^{m} \sum_{j=1}^{n}P \bigl(X_{i}e^{-R_{1}(\tau_{i})}>x, Y_{j}e^{-R_{2}(\eta_{j})}>y, M(t)=m, N(t)=n \bigr) \\ &\qquad{}-\sum_{m=M+1}^{\infty}\sum _{n=1}^{M}\sum_{i=1}^{m} \sum_{j=1}^{n}P \bigl(X_{i}e^{-R_{1}(\tau_{i})}>x, Y_{j}e^{-R_{2}(\eta_{j})}>y, M(t)=m, N(t)=n \bigr) \\ &\quad \equiv K_{11}(x,y;t)-K_{12}(x,y;t)-K_{13}(x,y;t). \end{aligned}$$

For $K_{11}(x,y;t)$, uniformly for all $t\in\varLambda_{T}$,

4.2 $$\begin{aligned} &K_{11}(x,y;t) \\ &\quad= \sum_{j=1}^{\infty}\sum _{i=1}^{\infty }P \bigl(X_{i}e^{-R_{1}(\tau_{i})}>x, Y_{j}e^{-R_{2}(\eta_{j})}>y, \tau_{i}\leq t, \eta_{j}\leq t \bigr) \\ &\quad= \sum_{j=1}^{\infty}\sum _{i=j+1}^{\infty}P \bigl(X_{i}e^{-R_{1}(\tau _{i})}>x, Y_{j}e^{-R_{2}(\eta_{j})}>y, \tau_{i}\leq t, \eta _{j}\leq t \bigr) \\ &\qquad{}+ \sum_{i=1}^{\infty}P \bigl(X_{i}e^{-R_{1}(\tau_{i})}>x, Y_{i}e^{-R_{2}(\eta_{i})}>y, \tau _{i}\leq t, \eta_{i}\leq t \bigr) \\ &\qquad{}+ \sum_{i=1}^{\infty }\sum _{j=i+1}^{\infty}P \bigl(X_{i}e^{-R_{1}(\tau_{i})}>x, Y_{j}e^{-R_{2}(\eta_{j})}>y, \tau_{i}\leq t, \eta_{j}\leq t \bigr) \\ &\quad \thicksim \int_{0}^{t} \int _{0}^{t}P \bigl(X^{*}e^{-R_{1}(u)}>x \bigr) P \bigl(Y^{*}e^{-R_{2}(v)}>y \bigr) \lambda(du, dv) \\ &\qquad{}+ \theta d_{1}d_{2}\sum_{i=1}^{\infty } \int_{0}^{t} \int_{0}^{t}P \bigl(X^{*}e^{-R_{1}(u)}>x \bigr)P \bigl( Y^{*}e^{-R_{2}(v)}>y \bigr) P(\tau_{i}\in du, \eta_{i}\in dv), \end{aligned}$$

where at the last step we used Lemma 3.6. In the following, we prove that $K_{12}(x,y;t)$ is asymptotically negligible compared with $K_{11}(x,y;t)$. For $K_{12}(x,y;t)$, by Lemma 3.2, uniformly for all $t\in\varLambda_{T}$,

4.3 $$\begin{aligned} &K_{12}(x,y;t) \\ &\quad \leq \bigl(1+ \vert \theta \vert b_{1}b_{2} \bigr)P \bigl(X^{*}e^{-\inf_{0\leq u\leq T}R_{1}(u)}>x \bigr) P \bigl(Y^{*}e^{-\inf_{0\leq v\leq T}R_{2}(v)}>y \bigr) \\ &\qquad{}\times EN(t)M(t)\mathbf{1}_{(N(T)\geq M)}. \end{aligned}$$

By (4.3) and Lemma 3.4, for $0<\epsilon<1$,

4.4 $$\begin{aligned} &\lim_{M\rightarrow\infty}\lim_{\min\{x,y\}\rightarrow\infty }\sup _{t\in\varLambda_{T}}\frac{K_{12}(x,y;t)}{\int_{0}^{t}\int _{0}^{t}P (X^{*}e^{-R_{1}(u)}>x, Y^{*}e^{-R_{2}(v)}>y ) \lambda(du, dv)} \\ &\quad\leq \lim_{\min\{x,y\}\rightarrow \infty}\frac{(1+ \vert \theta \vert b_{1}b_{2})P (X^{*}e^{-\inf_{0\leq u\leq T}R_{1}(u)}>x ) P (Y^{*}e^{-\inf_{0\leq v\leq T}R_{2}(v)}>y )}{P (X^{*}e^{-\sup_{0\leq u\leq T}R_{1}(u)}>x )P(Y^{*}\epsilon>y) P(e^{-\sup_{0\leq v\leq T}R_{2}(v)}>\epsilon)} \\ &\qquad \times \lim_{M\rightarrow\infty}\sup_{t\in\varLambda_{T}} \frac {EN(t)M(t)\mathbf{1}_{(N(T)\geq M)}}{\lambda(t,t)}=0. \end{aligned}$$

As above, as $M\rightarrow\infty$ and $\min\{x,y\}\rightarrow\infty $, we can prove $K_{13}(x,y;t)$ is also asymptotically negligible in comparison with $K_{11}(x,y;t)$. Hence, uniformly for all $t\in\varLambda_{T}$,

4.5 $$\begin{aligned} &K_{1}(x,y;t) \\ &\quad\thicksim \int_{0}^{t} \int_{0}^{t}P \bigl(X^{*}e^{-R_{1}(u)}>x, Y^{*}e^{-R_{2}(v)}>y \bigr) \lambda(du, dv)+\theta d_{1}d_{2} \\ &\qquad {}\times\sum_{i=1}^{\infty} \int_{0}^{t} \int_{0}^{t}P \bigl(X^{*}e^{-R_{1}(u)}>x, Y^{*}e^{-R_{2}(v)}>y \bigr) P(\tau_{i}\in du, \eta_{i}\in dv). \end{aligned}$$

Next we switch to deal with $K_{2}(x,y;t)$. Choose some $p>\max\{ J_{F}^{+}, J_{G}^{+}\}$. According to Lemma 3.2 and Lemma 3.3(1), uniformly for all $t\in\varLambda_{T}$,

$$\begin{aligned} &K_{2}(x,y;t) \\ &\quad\leq \sum_{m=1}^{M}\sum _{n=M+1}^{\infty }\sum_{i=1}^{m} \sum_{j=1}^{n}P \bigl(X_{i}e^{-\inf_{0\leq u\leq T}R_{1}(u)}>x/m, Y_{j}e^{-\inf_{0\leq v\leq T}R_{2}(v)}>y/n \bigr) \\ &\qquad{}\times P \bigl(M(t)=m, N(t)=n \bigr) \\ &\quad\leq C \bigl(1+ \vert \theta \vert b_{1}b_{2} \bigr)P \bigl(X^{*}e^{-\inf_{0\leq u\leq T}R_{1}(u)}>x \bigr) P \bigl(Y^{*}e^{-\inf_{0\leq v\leq T}R_{2}(v)}>y \bigr) \\ &\qquad{}\times E \bigl(M(t)N(t) \bigr)^{p+1}\mathbf {1}_{(N(T)\geq M)}, \end{aligned}$$

where *C* is a positive number. Following the proof of (4.4), we have

4.6 $$\begin{aligned} \lim_{M\rightarrow\infty}\lim_{\min\{x,y\}\rightarrow\infty}\sup _{t\in\varLambda_{T}}\frac{K_{2}(x,y;t)}{\int_{0}^{t}\int _{0}^{t}P (X^{*}e^{-R_{1}(u)}>x, Y^{*}e^{-R_{2}(v)}>y )\lambda(du, dv)}=0. \end{aligned}$$

Similarly to above, we can prove that

4.7 $$\begin{aligned} \lim_{M\rightarrow\infty}\lim_{\min\{x,y\}\rightarrow\infty}\sup _{t\in\varLambda_{T}}\frac{K_{3}(x,y;t)+K_{4}(x,y;t)}{\int_{0}^{t}\int _{0}^{t}P (X^{*}e^{-R_{1}(u)}>x, Y^{*}e^{-R_{2}(v)}>y )\lambda(du, dv)}=0. \end{aligned}$$

Substituting (4.5), (4.6) and (4.7) into (4.1), we find that (2.1) holds uniformly for all $t\in\varLambda_{T}$.

In what follows, we extend the uniformity of Eq. (2.1) to the whole interval *Λ*. By virtue of Lemma 3.10, for any $0<\epsilon<1$, there exists some constant $T_{0}$ such that, for any $k=1,2,3$, and $i=1,2,\ldots$ , the inequalities

4.8 $$\begin{aligned} &\int \int_{\varOmega_{k}(T_{0})}P \bigl(X^{*}e^{-R_{1}(u)}>x, Y^{*}e^{-R_{2}(v)}>y \bigr)\lambda(du,dv) \\ &\quad\leq \epsilon \int_{0}^{T_{0}} \int_{0}^{T_{0}}P \bigl(X^{*}e^{-R_{1}(u)}>x, Y^{*}e^{-R_{2}(v)}>y \bigr)\lambda(du,dv) \end{aligned}$$

and

4.9 $$\begin{aligned} &\int \int_{\varOmega_{k}(T_{0})}P \bigl(X^{*}e^{-R_{1}(u)}>x, Y^{*}e^{-R_{2}(v)}>y \bigr)P(\tau_{i}\in du, \eta_{i}\in dv) \\ &\quad \leq \epsilon \int_{0}^{T_{0}} \int_{0}^{T_{0}}P \bigl(X^{*}e^{-R_{1}(u)}>x, Y^{*}e^{-R_{2}(v)}>y \bigr)P(\tau_{i}\in du, \eta_{i}\in dv) \end{aligned}$$

hold for all sufficiently large *x* and *y*.

On the one hand, by Theorem 2.1, (4.8) and (4.9), for sufficiently large *x* and *y*, uniformly for all $t\in(T_{0}, \infty]$,

4.10 $$\begin{aligned} &P \Biggl(\sum_{i=1}^{M(t)}X_{i}e^{-R_{1}(\tau_{i})}>x, \sum_{j=1}^{N(t)}Y_{j}e^{-R_{2}(\eta_{j})}>y \Biggr) \\ &\quad \geq P \Biggl(\sum_{i=1}^{M(T_{0})}X_{i}e^{-R_{1}(\tau_{i})}>x, \sum_{j=1}^{N(T_{0})}Y_{j}e^{-R_{2}(\eta_{j})}>y \Biggr) \\ &\quad \geq (1-\epsilon) \biggl( \int_{0}^{t}- \int_{T_{0}}^{\infty} \biggr) \biggl( \int_{0}^{t}- \int_{T_{0}}^{\infty} \biggr)P \bigl(X^{*}e^{-R_{1}(u)}>x, Y^{*}e^{-R_{2}(v)}>y \bigr) \lambda(du, dv) \\ &\qquad{}+(1-\epsilon)\theta d_{1}d_{2}\sum _{i=1}^{\infty} \biggl( \int_{0}^{t}- \int_{T_{0}}^{\infty} \biggr) \biggl( \int_{0}^{t}- \int_{T_{0}}^{\infty} \biggr) P \bigl(X^{*}e^{-R_{1}(u)}>x, Y^{*}e^{-R_{2}(v)}>y \bigr) \\ &\qquad{} \times P(\tau_{i}\in du,\eta_{i}\in dv) \\ &\quad \geq (1-2\epsilon )^{2} \int_{0}^{t} \int_{0}^{t}P \bigl(X^{*}e^{-R_{1}(u)}>x \bigr)P \bigl(Y^{*}e^{-R_{2}(v)}>y \bigr) \lambda(du, dv)+(1-2 \epsilon)^{2} \\ & \qquad{}\times\theta d_{1}d_{2}\sum_{i=1}^{\infty} \int_{0}^{t} \int _{0}^{t}P \bigl(X^{*}e^{-R_{1}(u)}>x \bigr) P \bigl(Y^{*}e^{-R_{2}(v)}>y \bigr) P(\tau_{i}\in du, \eta_{i}\in dv). \end{aligned}$$

On the other hand, by Remark 2, (4.8) and (4.9), uniformly for all $t\in(T_{0}, \infty]$,

4.11 $$\begin{aligned} &P \Biggl(\sum_{i=1}^{M(t)}X_{i}e^{-R_{1}(\tau_{i})}>x, \sum_{j=1}^{N(t)}Y_{j}e^{-R_{2}(\tau_{j})}>y \Biggr) \\ &\quad \leq (1+\epsilon) \biggl( \int_{0}^{t}+ \int_{T_{0}}^{\infty} \biggr) \biggl( \int_{0}^{t}+ \int_{T_{0}}^{\infty} \biggr)P \bigl(X^{*}e^{-R_{1}(u)}>x \bigr)P \bigl(Y^{*}e^{-R_{2}(v)}>y \bigr) \lambda(du, dv) \\ &\qquad{}+(1+\epsilon)\theta d_{1}d_{2}\sum _{i=1}^{\infty} \biggl( \int_{0}^{t}+ \int_{T_{0}}^{\infty} \biggr) \biggl( \int_{0}^{t}+ \int_{T_{0}}^{\infty} \biggr) P \bigl(X^{*}e^{-R_{1}(u)}>x \bigr)P \bigl(Y^{*}e^{-R_{2}(v)}>y \bigr) \\ &\qquad{}\times P(\tau_{i}\in du, \eta_{i}\in dv) \\ &\quad \leq (1+2\epsilon)^{2} \int_{0}^{t} \int _{0}^{t}P \bigl(X^{*}e^{-R_{1}(u)}>x \bigr) P \bigl(Y^{*}e^{-R_{2}(v)}>y \bigr) \lambda(du, dv)+(1+2 \epsilon)^{2} \\ & \qquad{}\times\theta d_{1}d_{2}\sum_{i=1}^{\infty} \int_{0}^{t} \int _{0}^{t}P \bigl(X^{*}e^{-R_{1}(u)}>x \bigr) P \bigl(Y^{*}e^{-R_{2}(v)}>y \bigr) P(\tau_{i}\in du, \eta_{i}\in dv). \end{aligned}$$

Combining (4.10) and (4.11) and taking account into the arbitrariness of *ϵ*, we see that Eq. (2.1) holds uniformly for all $t\in(T_{0}, \infty)$. Hence, we complete the proof of Theorem 2.1.

### Proof of Theorem 2.2

For convenience, denote the right-hand side of (2.2) by $\phi _{\theta}(x,y;t)$. We first deal with the asymptotic upper bound of $\psi_{\max}(x,y;t)$. On the one hand, by Theorem 2.1, it follows that

4.12 $$\begin{aligned} \psi_{\max}(x,y;t) \leq P \Biggl(\sum_{i=1}^{M(t)}X_{i}e^{-R_{1}(\tau_{i})}>x, \sum_{j=1}^{N(t)}Y_{j}e^{-R_{2}(\eta_{j})}>y \Biggr)\thicksim\phi _{\theta}(x,y;t) \end{aligned}$$

holds uniformly for all $t\in\varLambda$. Then we discuss the asymptotic lower bound of $\psi_{\max}(x,y;t)$. For simplicity, write

$$\begin{aligned} Z_{i}= \int_{0}^{\infty}e^{-R_{i}(u)}\,du,\quad i=1,2. \end{aligned}$$

Notice that $Z_{1}$ and $Z_{2}$ are light-tailed. We can choose some slowly varying function $l(x)$ satisfying $0< l(x)\rightarrow\infty$, $l(x)/x\rightarrow0$, such that, for all $0\leq u,v\leq t$,

$$\begin{aligned} \lim_{x\rightarrow\infty}\frac{P (X^{*}e^{-R_{1}(u)}>x+l(x) )}{P (X^{*}e^{-R_{1}(u)}>x )}=1 \end{aligned}$$

and

$$\begin{aligned} \lim_{y\rightarrow\infty}\frac{P (Y^{*}e^{-R_{2}(v)}>y+l(y) )}{P (Y^{*}e^{-R_{2}(v)}>y )}=1. \end{aligned}$$

According to the definition of $\psi_{\max}(x,y;t)$, Theorem 2.1 and Fatou’s lemma, uniformly for all $t\in\varLambda$,

4.13 $$\begin{aligned} &\psi_{\max}(x,y;t) \\ &\quad\gtrsim P \Biggl(\sum_{i=1}^{M(t)}X_{i}e^{-R_{1}(\tau_{i})}>x+MZ_{1}, \sum_{j=1}^{N(t)}Y_{j}e^{-R_{2}(\eta_{j})}>y+MZ_{2} \Biggr) \\ &\quad \gtrsim P \Biggl(\sum_{i=1}^{M(t)}X_{i}e^{-R_{1}(\tau_{i})} >x+l(x), \sum_{j=1}^{N(t)}Y_{j}e^{-R_{2}(\eta_{j})}>y+l(y) \Biggr) \\ &\quad \thicksim \int_{0}^{t} \int _{0}^{t}P \bigl(X^{*}e^{-R_{1}(u)}>x+l(x) \bigr)P \bigl(Y^{*}e^{-R_{2}(v)}>y+l(y) \bigr) \lambda(du, dv)+ \theta d_{1}d_{2} \\ &\qquad{}\times\sum_{i=1}^{\infty} \int_{0}^{t} \int _{0}^{t}P \bigl(X^{*}e^{-R_{1}(u)}>x+l(x) \bigr)P \bigl(Y^{*}e^{-R_{2}(v)}>y+l(y) \bigr) P( \tau_{i} \in du, \eta_{i}\in dv) \\ &\quad \gtrsim \phi_{\theta }(x,y;t). \end{aligned}$$

A combination of (4.12) and (4.13) shows that (2.2) holds uniformly for all $t\in\varLambda$.

Now we begin to discuss the asymptotic behavior of $\psi_{\min}(x,y;t)$. It is not hard to see that

4.14 $$\begin{aligned} \psi_{\min}(x,y;t)=P(\vartheta_{1}\leq t)+P( \vartheta_{2}\leq t)-\psi_{\max}(x,y;t). \end{aligned}$$

Since for any fixed $T<\infty$, $N(T)<\infty$, there exists some $b>0$ such that

4.15 $$\begin{aligned} \sum_{j=1}^{\infty}P(\tau_{i}\in du, \eta_{j}\leq T)=\sum_{j=1}^{\infty}P \bigl(\tau_{i}\in du, N(T)\geq j \bigr)\leq bP(\tau_{i}\in du). \end{aligned}$$

According to (2.2) and (4.15), we have

4.16 $$\begin{aligned} &\lim_{\min\{x,y\}\rightarrow\infty}\limsup_{t\in\varLambda _{T}}\frac{\psi_{\max}(x,y;t)}{\int _{0}^{t}P(X^{*}e^{-R_{1}(u)}>x)\,d\lambda_{1}(u) +\int _{0}^{t}P(Y^{*}e^{-R_{2}(v)}>y)\,d\lambda_{2}(v)} \\ &\quad\leq \lim_{x\rightarrow\infty}\limsup_{t\in\varLambda_{T}} \frac{\sum_{i=1}^{\infty}\int_{0}^{t}P (X^{*}e^{-R_{1}(u)}>x ) (\sum_{j=1}^{\infty}P(\tau_{i}\in du, \eta_{j}\leq T) ) }{\sum_{i=1}^{\infty}\int_{0}^{t}P (X^{*}e^{-R_{1}(u)}>x )P(\tau_{i}\in du)} \\ &\qquad{}\times \bigl(1+ \vert \theta \vert \,d_{1}d_{2} \bigr) \lim_{y\rightarrow\infty}P \bigl(Y^{*}e^{-\inf_{0\leq v\leq T}R_{2}(v)}>y \bigr)=0. \end{aligned}$$

In terms of (4.14), (3.5), (3.6) and (4.16), we find that (2.3) holds uniformly for all $t\in\varLambda_{T}$.
